# Evaluation of the Relationship Between Isometric Exercise and Roll‐Over Tests and the Levels of Pregnancy‐Associated Plasma Protein A and Beta‐Human Chorionic Gonadotropin and Neutrophil–Lymphocyte Ratio in the Early Diagnosis of Preeclampsia

**DOI:** 10.1155/ogi/9393263

**Published:** 2026-01-31

**Authors:** Ozge Kozacioglu Ugan, Serap Ejder Apay

**Affiliations:** ^1^ Department of Midwifery, Faculty of Health Science, Ataturk University, Erzurum, 25100, Türkiye, atauni.edu.tr

**Keywords:** β-hCG, hypertension, midwife, NLR, PAPP-A, preeclampsia

## Abstract

**Objective:**

This study aimed to determine the relationship between isometric exercise test, roll‐over test, levels of PAPP‐A and β‐hCG, and NLO in the early diagnosis of preeclampsia.

**Materials and Method:**

The 580 pregnant women filled out the personal information forms and performed the roll‐over test and isometric exercise test.

**Results:**

The roll‐over test and isometric exercise test used in the diagnosis of preeclampsia had high levels of sensitivity and specificity and negative predictive values.

**Conclusion:**

Thus, roll‐over test and isometric exercise test, pregnancy‐associated plasma protein A levels, and neutrophil–lymphocyte ratio are useful parameters for the early diagnosis of preeclampsia.

## 1. Introduction

Typically, preeclampsia, which develops after the 20th week of pregnancy, is characterized by the presence of proteinuria or other clinical conditions in addition to hypertension. Recently, the prerequisites for the diagnosis of preeclampsia have undergone a significant change, and the presence of proteinuria is no longer a requirement for the diagnosis of preeclampsia. In patients without proteinuria, the condition is diagnosed as preeclampsia when the patient has at least one of the following symptoms, namely, thrombocytopenia, impaired liver function, newly emerging kidney dysfunction, pulmonary edema, and new‐onset cerebral and visual disturbances [1].

Hypertension is one of the important indicators in the diagnosis of preeclampsia; therefore, routine blood pressure measurement in the early stages of the pregnancy and in the second trimester is extremely important to determine when a patient may develop hypertension. Previous studies indicate that blood pressure measurements taken in the second trimester play an important role in determining the development of hypertension in the future [2–4]. Although significant advances have been made in the diagnosis and treatment of preeclampsia in the last 10 years, the etiopathogenesis of this condition has not been entirely explained thus far. Understanding the etiopathogenesis underlying this condition will enhance the prevention, diagnosis, and treatment for preeclampsia. In addition to hypertension and renal dysfunction, various other systems are affected by preeclampsia. The placenta is thought to be an important causative factor for preeclampsia because preeclampsia begins to resolve after the delivery of the placenta [5–8].

Preeclampsia, which is among one of the diseases associated with hypertension during pregnancy, is among the top three causes of maternal death worldwide [5, 9]. In 2018, the annual maternal mortality rate in Turkey was 13.6201 per 100,000 live births. The highest rate of maternal death of 24.0 was reported in Northeast Anatolia. The rate of maternal mortality during pregnancy, during childbirth, and postpartum maternal mortality was 0.06 in 2018 [10].

Understanding the risk factors associated with pregnancy, detecting these factors at an early stage, taking steps to minimize their negative effects on pregnancy, and protecting maternal and infant health are among the duties and responsibilities of a midwife [11]. Early diagnosis and appropriate management of hypertensive diseases during pregnancy are very important in the treatment and prevention of preeclampsia. The role of midwives is very important role in the management of such conditions during pregnancy [12–14].

Various tests, biomarker tests, and biochemical markers can be used to determine preeclampsia. Dr. Norman Gant, who developed the ROT, reported that more than 90% of the pregnant women who have a positive ROT developed hypertension caused by pregnancy and that pregnant women with a negative ROT have more than 90% chance of becoming normotensive for the remaining duration of their pregnancy [15–18]. High blood pressure seen during different exercise tests in pregnant women is thought to be related to hypertension caused by pregnancy. Degani et al. [19] reported a high diagnostic value of the IET. Although previous studies have examined the relationship between the levels of pregnancy‐associated plasma protein A (PAPP‐A) and beta‐human chorionic gonadotropin (β‐hCG) and pregnancy complications, the results obtained were not consistent. A decrease in the levels of PAPP‐A was identified as a significant predictor of preeclampsia in previous studies [20–23].

### 1.1. Aim

This study aimed to evaluate the relationship between IET, ROT, the levels of PAPP‐A and β‐hCG, and neutrophil–lymphocyte ratio (NLR) in the determination of preeclampsia.

## 2. Materials and Methods

### 2.1. Study Type

A prospective study was performed to determine the relationship between ROT, IET, the levels of PAPP‐A and β‐hCG, and NLR in the early diagnosis of preeclampsia.

### 2.2. Location and Time of Study

The study was conducted from June to October 2020 at the Gynecology Polyclinic in the Seyhan State Hospital Marsa Gynecology and Obstetrics Additional Service Building in Adana Province. The study was conducted in an outpatient clinic setting where approximately 100 pregnant women visited daily.

### 2.3. Study Population and Sample

The study population consisted of pregnant women who visited the relevant hospital between the specified dates. The study sample was selected from the study population using the random sampling method, and the study population consisted of pregnant women between 28 and 34 weeks of gestation who voluntarily agreed to participate and did not have any systemic diseases. A previously established formula was used to determine the sample size, and 580 pregnant women were included in the study.

### 2.4. Inclusion Criteria

Pregnant women, who routinely monitored the PAPP‐A levels, β‐hCG levels, and NLR, were between 28 and 34 weeks of gestation and did not have health problems or a previous history of hypertension.

### 2.5. Data Collection Methods and Tools

The data were collected using the personal information form. The personal information form consists of four parts. The first part consists of questions prepared by the researchers to determine the history of the pregnant women. Results of the ROT and IET were recorded in the second part of the form. The data regarding PAPP‐A and β‐hCG levels and NLR were recorded in the third part of the form. In the last part, women were personally called by phone with their permission and asked whether they had come to the hospital for blood pressure control. In addition, women’s medical diagnoses were checked in the system where their information was recorded. When the pregnant women visited the hospital to receive antenatal care, the purpose of the study was explained to them, and those who decided to participate in the study were taken to a suitable room in the hospital. The information in the first part of the personal information form was filled out; then, the pregnant woman was placed in the left lateral position for the ROT and rested for 10 min. In this study, the right arm was used to measure the blood pressure. To date, very few studies have focused on the arm used to measure blood pressure. A difference of up to 11 mmHg has been reported in blood pressure measured using the right (upper) arm compared with that measured using the left (lower) arm. This difference may be attributed to the 4^th^ or 5^th^ phases of Korotkoff sounds used in the evaluation of diastolic blood pressure [24]. Five phases of Korotkoff sounds were used for the evaluation of blood pressure in this study. Please check whether this sentence should be revised as “In this study, the blood pressure was measured in the left lateral position, thereby ensuring the comfort of the pregnant woman during the measurement.”

After the ROT was completed, the pregnant woman rested for another 10 min, and the IET was performed. Subsequently, the levels of PAPP‐A and β‐hCG and NLR in the pregnant women were obtained by an authorized person in the hospital in a way that would not disrupt the functioning of the hospital. Telephone calls were made to all women, depending on their estimated date of delivery, and the women were asked whether they developed preeclampsia during pregnancy, delivery, or the postpartum period. In addition, the patient registry system of the hospital was checked to determine whether the patient was diagnosed with preeclampsia during pregnancy, delivery, or the postpartum period. Patients were diagnosed with preeclampsia by a specialist physician according to ACOG guidelines (Diagnostic Criteria for Preeclampsia: Blood pressure, Proteinuria) [25].

### 2.6. ROT

The pregnant woman was placed in the left lateral position for 10 min, and then, the blood pressure was measured on the right arm. Subsequently, the pregnant woman was asked to return to the supine position; after 5 min in this position, the blood pressure was measured again on the same arm. During the change of positions, the pregnant woman was observed, and her well‐being was evaluated. In case of any discomfort, the test was terminated immediately. All participants completed the test successfully. The test was positive if an increase in diastolic pressure by ≥ 20 mmHg was observed during the transition from the left lateral position to the supine position, and the test was negative if the difference in the diastolic pressure was < 20 mmHg.

### 2.7. IET

After ROT, the patient rested for 10 min in the left lateral position, and blood pressure was measured again on the right arm. Then, the pregnant woman was asked to squeeze the inflated sphygmomanometer cuff by applying maximum force with her left hand. She was asked to squeeze the cuff with her left hand for 3 min and to remain constant at the maximum value determined. The blood pressure was measured again on the right arm after the exercise. The test was considered positive in case of an increase in diastolic blood pressure by ≥ 20 mmHg before and after the test.

### 2.8. Determination of the Levels of PAPP‐A and β‐hCG and NLR

After obtaining consent from the women, the values of PAPP‐A and β‐hCG levels and NLR recorded in the hospital database were taken without disrupting the hospital operations.

### 2.9. Data Analysis

SPSS was used to analyze the data. The number, percentage, mean, and *t*‐test in independent groups and Pearson’s correlation analysis were used in the analysis of the data. The following calculations were used for the sensitivity and specificity of the tests:

 
*A* = True‐positive patients
 
*B* = False‐positive patients
 
*C* = False‐negative patients
 
*D* = True‐negative patients
 
*A* + *B* = Total positives
 
*C* + *D* = Total negatives

Sensitivity is how many patients the test can detect (diagnose) among those who are actually ill.

 Sensitivity: *a*/(*a* + *c*) × 100

Specificity is how many patients the test can detect (rule out) among those who are actually healthy.

 Specificity: *d*/(*b* + *d*) × 100
 Positive predictive value (PPV): *a*/(*a* + *b*) × 100
 Negative predictive value (NPV): *d*/(*d* + *c*) × 100
 For ROT: Sensitivity = 45/(45 + 8) × 100 = 84.90%
 Specificity = 341/(341 + 186) × 100 = 64.70%
 PPV = 45/(45 + 186) × 100 = 19.48%
 NPV = 341 (341 + 8) × 100 = 97.70%
 For IET: Sensitivity = 49/(49 + 4) × 100 = 92.45%
 Specificity = 350/(177 + 350) × 100 = 66.41%
 PPV = 49/(49 + 177) × 100 = 21.68%
 NPV = 350 (350 + 4) × 100 = 98.87%

### 2.10. Ethical Considerations

Permissions were obtained from the ethics committee (B.30,2.ATA.0.01.00/183) and from the hospital where the study was conducted (96.172.664‐799); consent was obtained from the participants after informing them.

## 3. Results

The distribution of descriptive characteristics of the pregnant women is shown in Table 1.

**Table 1:** Distribution of the descriptive characteristics of the pregnant women.

**Characteristics**	***n***	**%**

Educational status		
Middle school graduate	141	24.3
High school graduate	367	63.3
University	72	12.4
Employment status		
Employed	180	31.0
Unemployed	400	69.0
Family type		
Nuclear	533	91.9
Extended	47	8.1
Result of a previous pregnancy		
First birth	212	36.6
Live birth	368	63.4
Problems during the previous pregnancy		
Yes	88	15.2
No	492	84.8
	$\overset{¯}{X}$ ± **SD**	

Age (years)	28.80 ± 6.11	
Age at marriage (years)	19.56 ± 2.03	
Age at first pregnancy (years)	20.46 ± 2.53	
Number of pregnancies	2.03 ± 0.93	
Number of live births	1.03 ± 0.93	
Pregnancy interval (year)	4.41 ± 2.03	
Pregnancy week	30.48 ± 1.84	
PAPP‐A value	0.81 ± 0.36	
β‐hCG	1.04 ± 0.46	
NLR	2.94 ± 0.68	

The comparison between the results of the ROT and IET and the PAPP‐A levels in pregnant women is shown in Table 2. Negative ROT and IET results were reported in 60.2% of the pregnant women, and 90.9% of them had negative medical diagnoses. The comparison of the ROT and IET results with the PAPP‐A values showed that the difference between the ROT and medical diagnoses and PAPP‐A levels was statistically significant (*p* < 0.001). However, the difference between IET and PAPP‐A levels was not statistically significant (*p* > 0.05).

**Table 2:** Comparison of ROT and IET and medical diagnosis results of pregnant women with PAPP‐A values.

Tests	*N*	%	PAPP‐A value	Test^∗^ and *p* value
ROT				
Positive	231	39.8	0.75 ± 0.34	*t* = −3.25
Negative	349	60.2	0.85 ± 0.36	**p** = 0.001
IET				
Positive	225	38.8	0.77 ± 0.37	*t* = −1.88
Negative	355	60.2	0.83 ± 0.35	*p* = 0.06
Medical diagnosis				
Positive	53	9.1	0.57 ± 0.43	*t* = −5.16
Negative	527	90.9	0.83 ± 0.34	**p** = 0.0001

*Note:* Values shown in bold represent statistical significance at *p* < 0.05.

^∗^ *t*‐test in independent groups.

The results of ROT and IET and β‐hCG levels are shown in Table 3. Negative ROT and IET results were reported in 60.2% of the pregnant women, and that 90.9% of them had negative medical diagnoses. The comparison between the ROT and IET results of the pregnant women and β‐hCG levels showed no statistical difference (*p* > 0.05). Additionally, no statistically significant difference was observed between the medical diagnoses of the pregnant women and their β‐hCG levels (*p* > 0.05).

**Table 3:** Comparison of ROT and IET and medical diagnosis results of pregnant women with β‐hCG levels.

Tests	*N*	%	β‐hCG value	Test^∗^ and *p* value
ROT				
Positive	231	39.8	1.05 ± 0.46	*t* = 0.43
Negative	349	60.2	1.03 ± 0.45	*p* = 0.66
IET				
Positive	225	38.8	1.02 ± 0.45	*t* = 0.67
Negative	355	60.2	1.05 ± 0.46	*p* = 0.50
Medical diagnosis				
Positive	53	9.1	0.99 ± 0.34	*t* = 0.78
Negative	527	90.9	1.04 ± 0.47	*p* = 0.43

^∗^ *t*‐test in independent groups.

The comparison of ROT and IET results with NLR showed a statistically significant difference (*p* < 0.001).

The relationship between the results of the ROT and IET and medical diagnoses and the PAPP‐A levels, β‐hCG levels, and NLR is shown in Table 4. The difference between the results of the ROT and PAPP‐A levels was not statistically significant (*p* < 0.05). However, a statistically significant difference was observed between the IET results and medical diagnoses and PAPP‐A levels (*p* > 0.001). The difference between the results of the ROT and IET and medical diagnoses and β‐hCG levels of the pregnant women was not statistically significant (*p* > 0.05). A statistically significant difference was observed between the results of the ROT and IET and medical diagnoses of the pregnant women and NLR (^∗^ *p* < 0.05, ^∗∗^ *p* < 0.001).

**Table 4:** Relationship between the ROT and IET and medical diagnoses and levels of PAPP‐A and β‐hCG and NLR.

**Tests**		**PAPP-A**	**β-hCG**	**NLR**

ROT	r^a^	0.078	−0.018	**−0.088** ^**∗**^
	*p*	0.060	0.666	**0.034**

IET	r^a^	**0.134** ^**∗∗**^	0.028	**−0.141** ^**∗∗**^
	*p*	**0.001**	0.500	**0.001**

Medical diagnosis	r^a^	**0.210** ^**∗∗**^	0.033	**−0.499** ^**∗∗**^
	*p*	**0.000**	0.431	**0.000**

*Note:* Values shown in bold represent statistical significance at *p* < 0.05.

^a^Pearson’s correlation analysis.

^∗^ *p* < 0.05.

^∗∗^ *p* < 0.001.

The status of development of preeclampsia and the results of ROT and IET are shown in Table 5. Of the 580 pregnant women, 231 (39.9%) were ROT (+) and 349 (60.1%) were ROT (−). Of the ROT (+) women, 45 (19.5%) developed preeclampsia, and 86 (80.5%) did not develop preeclampsia. Of the ROT (−) women, 8 (2.3%) developed preeclampsia, and 341 (97.7%) did not develop preeclampsia. Of the 580 pregnant women, 226 (38.9%) were IET (+), and 354 (61.1%) were IET (−). Of the IET (+) women, 49 (21.6%) developed preeclampsia, and 177 (78.3%) did not develop preeclampsia. Of the IET (−) women, 4 (1.1%) developed preeclampsia, and 350 (98.8%) did not develop preeclampsia. While 53 of 580 pregnant women developed preeclampsia, 527 of them did not.

**Table 5:** Status of preeclampsia development and results of ROT and IET.

Test results	Status of preeclampsia development		Total
	Yes	No	
ROT			
Positive	45 (7.8)	186 (32.1)	231 (39.9)
Negative	8 (14)	341 (58.8)	349 (60.1)
IET			
Positive	49 (8.4)	177 (30.5)	226 (38.9)
Negative	4 (0.7)	350 (60.3)	354 (61.1)
Total	53	527	580

The distribution of sensitivity, specificity, PPV, and NPV of the ROT and IET results in pregnant women is shown in Table 6. The ROT had a sensitivity of 84.90%, a specificity of 64.70%, a PPV of 19.48%, and a NPV of 97.70%. The sensitivity for the IET was 92.45%, the specificity was 66.41%, the PPV was 21.68%, and the NPV was 98.87%.

**Table 6:** Distribution of the sensitivity, specificity, positive predictive value, and negative predictive value of the ROT and IET.

	**ROT (%)**	**IET (%)**

Sensitivity	84.90	92.45
Specificity	64.70	66.41
Positive predictive value	19.48	21.68
Negative predictive value	97.70	98.87

## 4. Discussion

Here, the results of the relationship between the ROT and IET and the levels of PAPP‐A and β‐hCG and NLR, which are used in the early diagnosis of preeclampsia, have been discussed and compared with the results reported previously.

The difference between the ROT and medical diagnoses and PAPP‐A values was statistically significant. However, the difference between the IET and PAPP‐A values was not statistically significant (Table 1). ROT and IET are very important in the early diagnosis of preeclampsia, and an emphasis should be placed on performing these tests. Dündar et al. [26] reported a mean PAPP‐A multiple of the median (MoM) value of 0.74 ± 0.94 in the late preeclampsia group, similar to the results observed in this study, and 0.90 ± 0.51 in the unaffected group. In the study by Dündar et al., the values in the group with early preeclampsia were 0.29 ± 0.15, which was close to the values reported in the group with a positive medical diagnosis of 0.57 ± 0.43 [21]. Çakmak et al. [27] reported a mean PAPP‐A (MoM) value of 1.4 ± 0.7 in the group without preeclampsia and 0.9 ± 0.3 in the group with preeclampsia. Results of the relationship between medical diagnosis and PAPP‐A levels observed in this study were consistent with those reported in previous studies.

The comparison of the results of the ROT and IET and the β‐hCG levels was not statistically significant (*p* < 0.05) (Table 2). In two studies, Spencer et al. [21, 27, 28] reported results similar to those reported in this study in that no significant difference was observed between the development of preeclampsia and β‐hCG values. Çakmak et al. reported similar levels of β‐hCG (MoM) in women with and without preeclampsia; the β‐hCG levels were 1.4 ± 1.2 and 1.2 ± 0.7 in the group without and with preeclampsia, respectively. The results of the study by Çakmak et al. are similar to those reported in this study [27].

A significant relationship was observed between the results of the ROT and IET and NLR (*p* < 0.05, *p* < 0.001; Table 3). Wiedemann et al. [29] reported that the NLR was higher in women with preeclampsia and preterm labor than in women in the control group (Table 3). Madızlı et al. [26] reported that the NLR was 3.31 ± 1.22 in the group with early preeclampsia and 3.42 ± 1.04 in the group with late preeclampsia; these results were consistent with the results reported in this study.

No significant relationship was observed between the results of the ROT and IET and medical diagnosis and β‐hCG levels. A statistically significant difference was observed between the ROT and IET and medical diagnoses and NLR. The difference between the IET and medical diagnoses and PAPP‐A levels was statistically significant (Table 7). These results are similar to those reported previously.

**Table 7:** Comparison of ROT and IET and medical diagnosis results of pregnant women with NLR.

Tests	*n*	%	NLR	Test^∗^ and *p* value
ROT				
Positive	231	39.8	3.01 ± 0.77	***t* = 2.11**
Negative	349	60.2	2.89 ± 0.61	**p** = 0.03
IET				
Positive	225	38.8	3.06 ± 0.76	***t* = 3.41**
Negative	355	60.2	2.86 ± 0.61	**p** = 0.001
Medical diagnosis				
Positive	53	9.1	4.01 ± 0.74	***t* = 13.82**
Negative	527	90.9	2.83 ± 0.57	**p** = 0.0001

*Note:* Values shown in bold represent statistical significance at *p* < 0.05.

^∗^ *t*‐test in independent groups.

The results showed that 60.2% of the pregnant women had negative ROT and IET, and 90.9% of them had negative medical diagnoses. The sensitivity for the ROT was 84.90%, the specificity (selectivity) was 64.70%, the PPV was 19.48%, and the NPV was 97.70% (Table 5). Gant et al. [15, 30, 31] reported that the sensitivity and PPV for the ROT were 88% and 94%, respectively; Tekin et al. [30, 32] reported these values as 78% and 39%, respectively; Tekin et al. [30, 33] reported them as 50% and 47%, respectively. Tekin et al. [30, 34] reported that the ROT had a sensitivity of 75%, a specificity of 59%, a PPV of 21%, and a NPV of 94%. Gülcan reported that the first and the second ROT had sensitivity, specificity, PPV, and NPV of 43%, 85%, 46%, and 82% and 28%, 89%, 44%, and 80%, respectively [35].

The sensitivity of the IET was 92.45%, the specificity (selectivity) was 66.41%, the PPV was 21.68%, and the NPV was 98.87% (Table 5).

Degani et al. [19] reported that the IET has a sensitivity of 81%, specificity of 96.4%, PPV of 81%, and a NPV of 94%. Tekin et al. [30] reported that the IET had a sensitivity of 73%, a PPV of 98%, and a NPV of 94%, and that this test can be used in the early diagnosis of hypertension caused by pregnancy. Unlike in this study, Taş Tekin reported a sensitivity and specificity of 41.7% and 94.3%, respectively. In a recent meta‐analysis by Conde‐Agudelo et al. [36], the sensitivity of the IET was from 50% to 70%, and the specificity was approximately 85%. Of course, it is best to test for a serious condition like preeclampsia with new tests and biomarkers. However, in areas with poor transportation and poor weather conditions, or for women with financial difficulties, these tests may not always be available. In such cases, ROT and IET, which are easy to administer, cost‐effective, and harmless to both mother and baby, can be lifesaving. These tests will be easier to administer, especially considering the duties and responsibilities of midwives who work directly with pregnant women [16–18].

The results of ROT and IET reported in this study were different from those reported previously. The difference in the results observed in this study may be because the ROT and IET were performed in the later weeks of pregnancy. The IET should be cheap, easy, and noninvasive and has a high sensitivity to be accepted widely as a diagnostic method to facilitate therapeutic interventions. Although previous studies have reported varying results, generally moderate sensitivity values have been reported for the IET. In this study, the IET had a sensitivity ratio of 84.9%. These results indicate that the IET may be partly used to predict whether the patients will develop preeclampsia. The PPV of the test was 19.48%; thus, the IET can be used for detecting preeclampsia only in pregnant women who are normotensive. Thus, pregnant women with negative test results will not develop hypertension.

Thus, the sensitivity, specificity, and NPV for the ROT and IET used in the diagnosis of preeclampsia were high. While no significant relationship was observed between ROT and IET and β‐hCG levels, the PAPP‐A levels and NLR showed a significant relationship with the ROT and IET. The data obtained in this study were similar to those reported in previous studies.

## 5. Conclusions

The results of this study and those performed previously show that preeclampsia is an important health problem in Turkey as well as across the world. For early diagnosis and prevention of this condition, it is crucial for midwives who provide antenatal care to understand the risk factors underlying preeclampsia. The midwives providing antenatal care should follow a holistic approach, obtain detailed information regarding the medical history of the woman during antenatal care, perform blood pressure measurements accurately during follow‐up, and be up‐to‐date with the guidelines for the management of pregnant women diagnosed with preeclampsia. Midwives should be provided with in‐service trainings to effectively deal with this condition.

Further in‐depth studies on ROT, IET, and some biochemical values used in the early diagnosis of preeclampsia should be conducted in the future.

## Author Contributions

Serap Ejder Apay: conceptualization, writing–review and editing, writing–original draft, software, methodology, investigation, formal analysis, data curation, conceptualization, and supervision. Ozge Kozacioglu Ugan: writing, methodology, investigation, and formal analysis.

## Funding

This research was not supported by funding from any institution.

## Ethics Statement

All participants consented to be included voluntarily. This research was approved by the Ataturk University Research Ethics Committee (B.30,2.ATA.0.01.00/183), in accordance with the Declaration of Helsinki and current legislation.

## Conflicts of Interest

The authors declare no conflicts of interest.

## Data Availability

The data that support the findings of this study are available upon request from the corresponding author. The data are not publicly available due to privacy or ethical restrictions.
